# Beyond the time-on-task: an EEG-driven approach for effective physiological assessment of mental fatigue in simulated and real driving

**DOI:** 10.3389/fbioe.2025.1682103

**Published:** 2025-10-28

**Authors:** Andrea Giorgi, Vincenzo Ronca, Rossella Capotorto, Alessia Vozzi, Dario Rossi, Pietro Aricò, Gianluca Borghini, Marteyn Van Gasteren, Javier Melus, Marco Petrelli, Simone Sportiello, Carlo Polidori, Manuel Picardi, Fabio Babiloni, Gianluca Di Flumeri

**Affiliations:** ^1^ Department of Molecular Medicine, Faculty of Pharmacy and Medicine, Sapienza University of Rome, Roma, Italy; ^2^ BrainSigns srl, Rome, Italy; ^3^ Department of Computer, Automatic and Management Engineering, Faculty of Information Engineering, Computer Science and Statistics, Sapienza University of Rome, Roma, Italy; ^4^ Department of Anatomical, Histological, Forensic and Orthopedic Sciences, Sapienza University of Rome, Roma, Italy; ^5^ Instituto Tecnológico de Castilla y León (ITCL) Technology Centre, Burgos, Spain; ^6^ Department of Civil Engineering, Computer Science and Aeronautical Technologies, Roma Tre University, Roma, Italy; ^7^ Department of Enterprise Engineering, University of Rome Tor Vergata, Rome, Italy; ^8^ Italian Association of Road Safety Professionals (AIPSS), Rome, Italy; ^9^ European Driving School Association, Brussel, Belgium; ^10^ Department of Physiology and Pharmacology, Faculty of Pharmacy and Medicine, Sapienza University of Rome, Roma, Italy; ^11^ College of Computer Science and Technology, Hangzhou Dianzi University, Hangzhou, China

**Keywords:** mental fatigue, physiological assessment, EEG, simulated driving, real driving, time-on-task, heart activity, ocular activity

## Abstract

**Introduction:**

Fatigue is a major factor contributing to road accidents, and extensive research has focused on its physiological and behavioral characterization. Due to safety and economic constraints, studies on driving fatigue are commonly conducted in simulated environments, where fatigue is typically induced through prolonged tasks and assessed using a Time-on-Task (ToT) approach. However, ToT-based labeling may not accurately reflect individual variations in fatigue onset.

**Methods:**

This study compared fatigue onset in matched simulated and real driving conditions by evaluating two labeling approaches: the traditional ToT-driven method and a novel physiology-driven method based on electroencephalographic (EEG) parameters. Experimental periods of Low and High Fatigue were defined using both approaches, and physiological and behavioral responses were analyzed through ocular and cardiac activity.

**Results:**

When using the ToT-driven approach, no significant differences emerged between low and high fatigue periods across the two environments. In contrast, the EEG-driven labeling revealed clear physiological responses to fatigue onset, as evidenced by changes in ocular and heart activity.

**Discussion:**

The findings demonstrate that the method used to define fatigue substantially influences the detection of fatigue onset. The results highlight the importance of physiology-based labeling for capturing individual fatigue dynamics and provide novel insights into how fatigue manifests differently in simulated and real driving contexts.

## Introduction

Mental fatigue has a dramatic impact on road safety. It is estimated that up to a third of road accidents might be caused by fatigue while driving (World Health Organization, 2023; Zwahlen et al., 2016). Its impact is not limited to road accidents. Also in the maritime (Fan and Yang, 2024; Grech, 2016; Lützhöft et al., 2011) and aviation (Caldwell, 2005; Wilson et al., 2007) field, fatigue has long been recognized as one of the main factors contributing to fatal and non-fatal accidents. However, while the impact of fatigue in maritime and aviation contexts is limited due to the relatively small number of individuals operating in those domains, its impact on road safety is amplified by the large number of people who drive on a daily basis (Directorate-General for Mobility and Transport European Commission, Oxford Research, Panteai, Tetra Tech, TIS, 2023). Indeed, with an approximative estimate of 300 million drivers in Europe (ACEA’s, Vehicles on European Roads, 2024), around 20% of drivers have reported driving at least once while being too tired to keep their eyes open (European Commission, 2021). From this, it is easy to imagine the huge impact of fatigued driving on road safety globally.

The detrimental effects of fatigue reside in the fact that it can alter human performance to a point where the individual is not able to perform a task, i.e., in this case driving a vehicle, adequately and therefore safely (Behrens et al., 2023). Fatigue can be divided into muscular fatigue and mental fatigue (Behrens et al., 2023), which can be modulated by both internal (age, gender, and others) and external factors (duration, intensity, and others). The first can be defined as a reduction of voluntary force capacity and it is only marginally involved in fatigue-related reduction of performance in operative contexts such as while driving. The second, cognitive or mental fatigue, can be defined as a decline in an objective cognitive performance measure (Behrens et al., 2023) (such as reaction time or accuracy). This decline can be observed both during the execution of a task as well as after the task is completed, depending on both internal and external factors (Wang et al., 2014). A well-established interpretation of fatigue-related performance decline suggests that during prolonged tasks, the required effort outweighs the perceived reward, leading to reduced motivation and, consequently, impaired performance (Benoit et al., 2019). Supporting this interpretation, Hopstaken and colleagues observed that the decline in cognitive performance due to time-on-task (ToT) was reversed when task rewards were increased (Hopstaken et al., 2015). Also in the case of prolonged driving, the associated fatigue has been observed to reduce driving performance (Sagaspe et al., 2008). In this context, fatigue-related declines in cognitive performance can have severe, and potentially tragic, consequences, such as, for instance, a failure to brake in time when a pedestrian is crossing the road. Despite the fact that there is a broad consensus on the impairing effects of fatigue, there is not a unitary definition of this construct, and it is often used interchangeably with terms like tiredness and drowsiness. In his report on road safety, Phillips (Phillips, 2014) listed several definitions of fatigue used in literature, highlighting the multi-component nature of the fatigue construct. In the present manuscript, we refer to fatigue as defined by Craig and colleagues (Craig et al., 2006): “a psychophysiological state that occurs when a person is driving and feeling tired or drowsy, to the extent that they have reduced capacity to function, resulting in performance decrements and negative emotions and boredom as they attempt to stay awake during the task”. Considering both the physiological and psychological dimensions of fatigue offers important operational advantages. Defining fatigue as a physiological state enables robust and objective measurement through various methodologies. Simultaneously, acknowledging its psychological dimension accounts for the subjective experience of fatigue, where individuals may feel fatigued even if they have not yet reached a point of impaired task performance (Phillips, 2015). Physiological and subjective measures are indeed among the most used approaches in fatigue-related research. In terms of fatigue subjective assessment, users are asked to rate their perception of fatigue through questionnaires. Researchers developed several questionnaires to collect subjective data. These questionnaires may be either self-declared or filled out by an external user reporting the severity of observed sleepiness or fatigue. For the self-declared, one of the most adopted is the Karolinska Sleepiness Scale (Kaida et al., 2006) (KSS), which asks drivers to rate their mental state on a scale ranging from alertness to sleepiness. A similar approach is adopted by the Stanford Sleepiness Scale (Shahid et al., 2012). A further self-reported questionnaire that focuses specifically on the concept of fatigue, rather than sleepiness, is the Chalder Fatigue Scale (Cella and Chalder, 2010) (CFS). It requires participants to rate the severity of fatigue-related symptoms, which are categorized into two domains: physical and mental. On the other hand, physiological signals coming from the user are collected and analyzed in order to assess its internal state. In recent years, researchers have developed a large body of knowledge regarding the physiological characterization of fatigue and sleepiness (Geldreich, 1939; Thiffault and Bergeron, 2003; Brandt et al., 2004; Fan et al., 2007; Oron-Gilad and Ronen, 2007; Bundele and Banerjee, 2009; Danisman et al., 2010; Borghini et al., 2012; Kong et al., 2015; Fu et al., 2016; Awais et al., 2017; Kong et al., 2017; Choi et al., 2018; Fujiwara et al., 2019; Ghourabi et al., 2020; Doudou et al., 2020; Bafna and Hansen, 2021; Arefnezhad et al., 2022; Di Flumeri et al., 2022). It has been observed that the fatigued state is associated with changes in the physiological responses of the drivers. Fatigue has been linked to a change in brain activity. Using Electroencephalography (EEG), researchers have demonstrated that low-frequencies EEG bands are characteristic of a fatigued state. Particularly, an increase in Alpha (Di Flumeri et al., 2022; Fujiwara et al., 2019), Theta, and Delta (Nguyen et al., 2017; Arefnezhad et al., 2022) rhythms has been observed during fatigue, as well as the appearance of rapid oscillatory phenomena, known as “spindles,” in the theta and alpha bands themselves (Houshmand et al., 2021; Simon et al., 2011). An additional source of information to understand fatigue is represented by ocular activity, which can be studies either with videorecording or by electrodes which capture electrical oscillations caused by eye dynamics (Electrooculography, EOG). It has been found that an increase in blink rate (Papadelis et al., 2007) (EBR), blink duration (Danisman et al., 2010; Shekari Soleimanloo et al., 2019) (EBD), and percentage of eye closure (Sommer and Golz, 2010) (PERCLOS) is associated with fatigue. The peripheral nervous system provides also relevant information to understand fatigue and drowsiness. Heart activity is particularly studied in this context since it is particularly easy to collect this data using non-intrusive and wearable devices. Both electrical, Electrocardiography (ECG), and optical signals, Photopletysmography (PPG), are commonly used to assess cardiac correlates of fatigue. The time-varying distance between consecutive heartbeats, i.e., the heart rate variability (HRV), has been observed to be linked to drowsy state, where HRV was found to decrease in sleepiness compared to alertness (Alaimo et al., 2020; Fujiwara et al., 2019). The effort spent to understand physiological and psychological markers of fatigue is aimed at developing solutions to mitigate or to prevent fatigue-related impairments which can impact on users’ safety, such as the development of Advanced Driver Assistance Systems (Doudou et al., 2020) (ADAS), equipping the modern vehicles and aimed to detect drowsiness and to alert drivers.

Nevertheless, while certain mental states such as workload, stress, and attention can be experimentally induced or modulated in intensity through the use of specific cognitive tasks, mental fatigue is more difficult to elicit in a controlled manner. In most cases, it can only be allowed to emerge spontaneously over time, with a high degree of inter-individual variability (Ackerman, 2011). To induce fatigue, researchers usually ask participants to perform extensive driving tasks lasting 1 hour or more, both in simulated and real driving (Fu et al., 2016; Joshi et al., 2020; Vicente et al., 2016). Two tasks’ features are usually manipulated by researchers to induce fatigue: difficulty and duration. The difficulty represents the amount of cognitive resources needed to perform the task. The higher the difficulty, the higher, or the sooner, the mental fatigue occurrence. On the other hand, the duration represents the extension of cognitive demand. Researchers often rely on task duration to induce fatigue, an effect known as time-on-task (ToT) (Ackerman and Kanfer, 2009; Lim et al., 2010; Hopstaken et al., 2015; Behrens et al., 2023). Performance decreasing with ToT is considered as the most common consequence of fatigue increasing. Although ToT approach represents a well-recognized method to induce mental fatigue, there are some results which conflict with the decrease in performance due to increasing ToT (Hockey, 2011; Nakagawa et al., 2013). These findings have been attributed to a learning effect taking place during the execution of a task, as well as by a reduced motivation in attending the task, with a redirection of the effort and consequent decrease in performance. Considering these findings, the ToT approach may not always be the most reliable method for defining fatigue levels in prolonged tasks. In particular, it relies on the assumption that all individuals experience fatigue at the same point in time, typically at the end of the task. Therefore, according to this assumption, the last part of the experiment is taken as representative of a fatigued condition, in contrast with the beginning considered representative of maximum alertness. However, this may not accurately reflect individual variability in fatigue onset and progression. A more robust approach to “label” fatigue severity along an experiment would involve the use of an objective, user-tailored measure of fatigue, as an alternative to the traditional ToT method. This approach would offer several key advantages: it accounts for inter-individual variability; avoids *a priori* assumptions about the presence of fatigue; eliminates the need to rely on self-reported measures, which may themselves influence participants’ perception of fatigue; enables the investigation of fatigue onset in addition to more advanced fatigue states; and provides a continuous and objective reference for training AI models with a validated ground truth. Physiological information represents optimal candidate to detect fatigue. As reported above, researchers already adopted physiological signals, from brain to ocular and heart activity, to assess the fatigued state. Among them, brain activity appears to be the most effective candidate for detecting the onset of fatigue. It is indeed plausible that changes in mental state first manifest in the central nervous system before becoming observable in peripheral physiological responses (Giorgi et al., 2023).

Besides this mere methodological approach, another concern when dealing with driving fatigue is the experimental setting. To minimize risks for participants, most experiments are conducted using driving simulators. This approach relies on the assumption that drivers’ physiological and behavioral responses in simulated environments closely mirror those observed in real driving conditions. However, this assumption does not fully consider that on-road driving and driving simulation differ substantially, particularly due to drivers’ awareness of the real-world consequences of errors during actual driving. Despite growing adoption of driving simulators, comprehensive and robust investigations between simulated and real driving remain limited (Engström et al., 2005; Li et al., 2013; Le et al., 2020; Shechtman et al., 2009). Most of these studies focused on comparing simulated and real driving in normal conditions (i.e., unimpaired) as well as under the influence of a high cognitive load and distraction, leaving a lack of robust evidences regarding the validity of driving simulator for studying mental fatigue while driving.

In line with this perspective, in the present study the primary aim was to compare two different approaches in assessing mental fatigue. We investigated fatigue-related physiological correlates obtained with both a ToT-driven approach and with an EEG-driven approach. With ToT-driven approach, the initial and the final part of a low-demanding driving task, lasting 45 min, were considered respectively as low fatigue and high fatigue periods. With the EEG-driven approach, an EEG-derived index to detect drowsiness (Di Flumeri et al., 2022) was used to label epochs as low fatigue and high fatigue based on the minimum and maximum levels of drowsiness detected through EEG analysis. The choice to adopt an EEG-derived index for drowsiness relies on the interpretation of fatigue as “a psychophysiological state that occurs when a person is driving and feeling tired or drowsy” (Craig et al., 2006). A secondary aim was to explore potential differences in the manifestation of fatigue between simulated and real driving environments. Both subjective and physiological responses during the driving task were compared across the two conditions. The study addressed the following research questions (RQs):

1. RQ1: Do physiological correlates of fatigue onset differ when assessed using a ToT-driven approach versus an EEG-driven approach? If yes, which of them promote sensitivity towards physiological responses linked to the investigated phenomenon, i.e., mental fatigue? Our hypothesis is that using an EEG-driven approach will take into account the inter-individual variability in fatigue development, increasing the difference between low and high fatigue periods. Therefore, a higher significant effect is expected for the EEG-driven approach compared to the ToT-driven approach.
2. RQ2: Do subjective and physiological correlates of fatigue onset differ between simulated and real driving conditions? Given the exploratory nature of RQ2, no *a priori* hypotheses were formulated.

To answer these questions, we collected and analyzed data through a prolonged and monotonous driving protocol, identical across both simulated and real-world environments. The focus on the onset of fatigue, rather than its more severe stages, stems from the rationale that early intervention is more effective in preventing the detrimental effects of fatigue. Accordingly, we aimed to compare the ability of a ToT-driven approach and an EEG-driven approach to detect the onset of mental fatigue.

## Methods

### Participants

A total of twenty-eight professional male drivers (n = 28, 28 M) were recruited to take part in the study. For the present manuscript, a subset of these participants was considered. This consisted of fourteen drivers (n = 14, 14 M) with an average age of 30.57 yo (±10.23 SD). The choice of reducing the number of participants considered for the analysis is based on two criteria: first, participants with corrupted data (at least one among brain, ocular, or heart activity) were excluded from the analysis (excluded n = 4); second, participants experiencing motion sickness due to the simulated environment were excluded from the analysis (excluded n = 10). The inclusion criteria for participation in the study were: possession of a valid driver’s license; normal or corrected-to-normal vision; no use of psychoactive drugs; and absence of any diagnosed mental illness.

The experiment was conducted following the principles outlined in the Declaration of Helsinki of 1975, as revised in 2008, and it was approved by the Sapienza University of Rome and Roma Tre Ethical Committee.

### Experimental protocol

**FIGURE 1:**
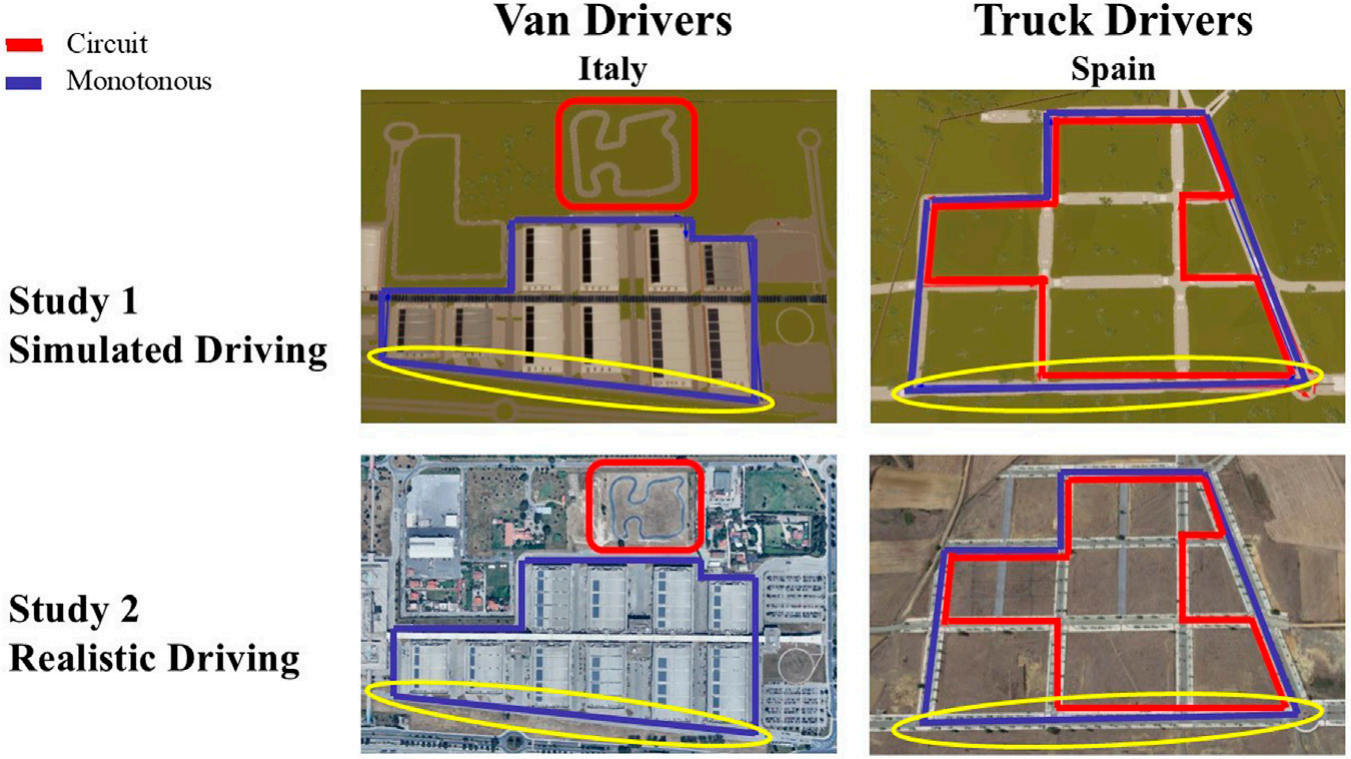
Simulated and Real environments adopted for the driving study. Experiments took place in two locations, Italy (left) and Spain (right). The real-world environments (Study 2, bottom) were accurately reproduced in the simulator software (Study 1, top). Both simulated and real-world driving consisted in a high-demanding driving task (red) and low-demanding driving task (blue). Analysis was performed on the longest straight part (circled in yellow) in order to reduce interindividual variability when comparing physiological correlates of fatigued driving.

Driving experiments were conducted in two locations: Rome (Italy), and Leon (Spain), where different professional drivers were recruited: respectively, van drivers and truck drivers. Indeed, the location selected in the two cities provided distinct driving environments which best met the road requirements needed to perform experiments with the two different vehicles. In Italy, where van drivers were recruited, the location “Fiera di Roma” was selected. It is a suburban private complex in the periphery of Rome, prohibited to public traffic. This location was chosen because it was possible to rent the entire location for a few days and close it to the public. It was constituted by two road infrastructures (Figure 1, left, red and blue). The first, in red, was a short racetrack, presenting a few straights and a high number of curves with various steering angles, thus resulting in a complex driving environment. The second, in blue, presented few ninety-degrees curves and long straights, so it was considered an easy driving environment. As described below in this paragraph, these characteristics were used to design two different driving tasks along the experiment, a high-demanding and a low-demanding one (Figure 1, left, respectively in red and blue). Likewise, with the same aim of selecting a controlled and safe environment for the tests in Spain with truck drivers, it was chosen an abandoned urbanization in Villatoldanos, Leon. Despite being a public road, this area does not present traffic. Differently from “Fiera di Roma” exhibition place, this location is constituted by a single road infrastructure with various intersections. This feature was exploited to design two different driving paths along the same road infrastructure. This was necessary to design two different tasks in terms of difficulty (Figure 1, right, in red the high-demanding task and blue the low-demanding one), as done for the Italian location. Once the locations for the realistic driving task were selected, they were precisely recreated in a virtual environment to ensure that participants experienced identical scenarios. The only difference was that in one case participants drove in a Simulated environment (Figure 1, Study 1), while the other took place in a Real setting (Figure 1, Study 2). All participants performed first the Simulated driving and then, after 5–6 months, the Real driving, in fixed order. This choice was made because the ethical committee required to reduce at minimum the number of participants for the Real driving due to safety concerns. Therefore, being aware of the possibility of participants withdrawing because of motion sickness due to simulated driving, and motion artifacts interfering with optimal data collection, we preferred to perform first the Simulated driving. Once we had a sufficient number of high-quality data, we were able to recruit these same participants also for the Real driving, which was expected to induce less motion sickness and consequently result in reduced data loss.

**FIGURE 2:**
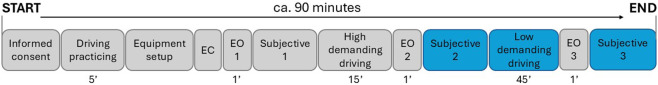
Both simulated and real-driving experiments lasted approximately 90 min from participants arrival to the end of the experiment. Data analysis in the present manuscript focused on the low-demanding driving task, which lasted 45 min. The assumption, confirmed by comparing subjective reports before (“Subjective 2”) and after the driving task (“Subjective 3”), was that in a 45-min task a moderate level (i.e., onset) of fatigue would be induced.

Independently from the locations, the protocol adopted for both Simulated and Real driving was identical both in Italy and Spain and therefore it was described here once, and it is shown in Figure 2. The experiment lasted around 90 min, from the arrival of participants at the location to the end of the test. At their arrival, inclusion criteria to take part in the study were checked, and then participants were provided with the informed consent form to sign, in order to accept participation in the study. After this preliminary phase, the devices for data collection were set up.

The simulated driving setup consisted of a real car seat, in a cockpit with a steering wheel, manual gearshift, and pedals, as well as a three-monitor display providing a 160° field of view. Participants were instructed to sit comfortably and were given the opportunity to practice with the simulator. A 5-min training session was conducted on the Monotonous scenario to ensure that all participants began the driving task with a consistent level of familiarity with the simulated environment. The realistic driving setup involved a Renault Trafic van. Similarly, participants were allowed to familiarize themselves with the vehicle by driving for 5 min on the same low-demanding task, ensuring a uniform level of confidence across all participants before starting the experiment. After the familiarization phase, the data collection started. While sitting on the seat, participants were instructed to close their eyes and relax for 1 min (“Eyes Closed” condition, EC). The same procedure was performed again but participants were asked to open their eyes and to look in front of them (“Eyes Open” condition, EO1). Participants were then asked to rate their level of fatigue and drowsiness before starting the high-demanding driving task. To do this, the KSS and the CFS questionnaires were provided (described below in this section). After this, the driving session started. Participants were asked to drive first in the high-demanding task and then in the low-demanding driving tasks, in fixed order. The high-demanding task lasted 15 min. It was aimed at challenging the mental resources of participants to increase the chance of inducing fatigue in the following easy and monotonous driving task. Participants were asked to drive as fast as possible to increase the difficulty of the task (Garcia et al., 2010; Thiffault and Bergeron, 2003). After the first driving task, a new “Eyes Open” condition was performed (“EO2”) and questionnaires were provided. Then, participants attended the low-demanding driving task, which lasted 45 min. This task was designed to be easy and repetitive, in contrast with the previous high-demanding driving task. The switch from a high-demanding to a low-demanding and easy driving task it is supposed to increase the chance of experiencing fatigue while driving (Garcia et al., 2010; Thiffault and Bergeron, 2003). After the second driving task, the last “Eyes Open” condition was performed (EO3). A representation of the experimental protocol adopted to conduct the experiment is provided in Figure 2. Considering that during the first high-demanding driving task participants were instructed to drive as fast as possible, leading to a high number of artifacts both in EEG and other signals, the assessment of fatigue described in the present paper was focused on the data related to the low-demanding task (Figure 2), in which data was expected to be less impacted by artifacts rejection. Therefore, in the present paper the collection of subjective data before and after the low-demanding driving task were referred to “Before task” and “After task” (respectively named “Subjective 2” and “Subjective 3” in Figure 2).

Fatigue was supposed to arise as a consequence of the experimental task, however, in order to promote the fatigue onset, experiments took place in the afternoon. In fact, literature reports that the daily moments in which users are more likely to experience fatigue while driving are during the afternoon and during the night (Zhang et al., 2023). As described in the Participants section, some of the participants experienced motion sickness during the tests. This is common in simulated driving experiments therefore, before starting to drive, the concept of motion sickness was briefly introduced to the participants, together with main symptoms. They were instructed to pay attention to eventual symptoms and if necessary, they could ask for a pause or withdraw from the experiment with no consequences on their reward.

### Subjective assessment

Two questionnaires were provided at participants’ arrival and after each driving task: the KSS (Kaida et al., 2006) and the CFS (Cella and Chalder, 2010), aimed to assess respectively the level of drowsiness and fatigue of participants. The choice of providing both questionnaires might be interpreted as redundant measures. This choice was made because, even if fatigue and drowsiness are distinct phenomena in terms of a conceptual and neurophysiological point of view, they are often considered two degrees of intensity on a continuous scale ranging from alertness to sleepiness (Kamran et al., 2019). In this view, the choice to ask the participants to fill out both questionnaires was made because, being contiguous phenomena, they are often hard to distinguish between each other, especially if considering the poor sensitivity of subjective measures. Questionnaires were provided according to the mother language of participants, therefore experimenters provided them translated in Italian and Spanish. Participants were instructed to fill in the questionnaires autonomously after their explanation.

#### Karolinska sleepiness scale

KSS (Kaida et al., 2006) requires participants to rate their current state of sleepiness on a scale from 1 (extremely alert) to 9 (extremely sleepy–fighting sleep).

#### Chalder fatigue scale

CFS questionnaire (Cella and Chalder, 2010) requires participants to answer several questions about fatigue-related symptoms on a scale from 0 (none) to 3 (very high). In the original form, CFS questions refer to two different dimensions called “physical symptoms” and “mental symptoms.” Considering the aim of the present investigation (i.e., mental fatigue), only the questions related to this dimension were used.

### Neurophysiological assessment

Several neurophysiological signals have been considered to characterize fatigue onset. Specifically, Electroencephalography (EEG), Electrooculography (EOG), and Photoplethysmography (PPG) measures have been recorded during the driving experiment. Initially, also Electrodermal Activity (EDA) was considered. When analyzing the data, a high number of motion artifacts were found in EDA signal, being the sensors placed on the wrist of the non-dominant hand. Since the intention was to have a consistent dataset for each measure and each participant, we decided to discard this signal in order to maintain an adequate number of participants for the remaining signals (EEG, EOG, and PPG). In any case, a preliminary analysis (not published here) on the available data (n = 17 showed no significant impact of fatigue onset on EDA-derived components, Skin Conductance Level and Response.

#### Electroencephalographic assessment

The Mindtooth wearable device (BrainProducts GmbH, Germany; https://mindtooth-eeg.com) was used to collect EEG data while driving. It is a high-grade EEG recording headset developed and validated during the Mindtooth Project (Sciaraffa et al., 2022) (GA 950998). It consists of 8 Ag/AgCl electrodes, holding water-based sponges in order to avoid the use of gel, placed according to the 10-10 International System (AFz, AF3, AF4, AF7, AF8, Pz, P3, and P4), in addition to a ground and a reference electrode placed on the mastoids. The sampling frequency was set at 125 Hz.

To avoid interference due to the mainline electrical power, a 50 Hz notch filter was applied. The EEG signal was then band-pass filtered [low-pass filter cut-off frequency: 40 (Hz), high-pass filter cut-off frequency: 2 (Hz)]. After filtering, the o-CLEAN (Ronca et al., 2024) algorithm was applied to detected EEG epochs affected by eyeblink artifacts. For other kinds of artifacts, dedicated algorithms of the EEGLAB toolbox for artifacts detection and rejection (Brunner et al., 2013) were applied using MATLAB. In particular, the processed EEG signal was divided into epochs of 1 s and a threshold criterion has been applied to reject artefactual data. It consists in labelling as artifacts all the epochs exceeding ±200 µV. All the epochs labelled as artifacts were later removed in order to have a clean EEG signal for the analysis. It was estimated a total of 3.15% of data loss due to artifacts rejection both in EEG and EOG (EEG-derived) signals (data rejection percentage per channel, mean ± standard deviation): AFz = 2.64 ± 2.84, AF3 = 3.14 ± 3.46, AF4 = 2.83 ± 3.12, AF7 = 2.99 ± 3.01, AF8 = 4.03 ± 4.86, Pz = 3.22 ± 4.38, P3 = 3.09 ± 4.18, and P4 = 3.25 ± 4.48). Independent Component Analysis (ICA) was not applied during artifact removal, as the preprocessing pipeline was designed to minimize data loss while preserving the neurophysiological signals of interest. Instead, artifact handling relied on alternative procedures considered more suitable for the characteristics of the present dataset and experimental design.

The clean signal was used to compute the Global Field Power (GFP) of the Alpha band over the parietal electrodes. The GFP was adopted as it provides a measure of cortical brain activity and has the advantage of representing, in the time domain, the degree of synchronization within a specific cortical region of interest in a given frequency band (Skrandies, 1990). The Alpha band was adopted because the present work aims at using a previously validated EEG index for mental drowsiness which is based on increased Alpha activity on parietal region (Di Flumeri et al., 2022). According to Klimesch (Klimesch, 1999), for each participants the Alpha band was defined using the Individual Alpha Frequency (IAF), estimated from the peak frequency within the alpha band during the ‘EC’ resting state condition, when participants were sitting with their eyes closed on the simulator seat at the beginning of the experiment. This procedure was adopted since the alpha peak is prominent when an individual is resting (Klimesch, 2012). Then, an EEG “strict” Alpha was defined as IAF Alpha = (IAF - 1): (IAF +1) Hz. This definition of the Alpha band is more restrictive (thus “strict”) compared to most of the Alpha band definitions that can be found in scientific literature, which is (IAF - 2): (IAF +2) Hz. This approach was adopted to avoid the impact from closer EEG frequency bands (Theta and Beta) variations on the Alpha band, as proposed in the original study (Ronca et al., 2022). The GFP was calculated over all the EEG parietal channels for each epoch using a Hanning window of the same length of the considered epoch (1 s length, which means 1 Hz of frequency resolution).

The EEG was used to compute a Mental Drowsiness index (Di Flumeri et al., 2022) (MDrow) every s. To improve stability, the index was segmented using 60-s windows with a 15-s overlap, thus reducing the temporal resolution from 1 s to 15 s.

For each segment, the MDrow index was calculated as follows:

$$MDrow=\frac{\text{Alpha}\;\text{GFP}\;\left[\text{Task}\right]}{\max\left(\text{Alpha}\;\text{GFP}\left[\text{Rest}\right]\right)}$$

Where the low-demanding task was considered as the *Task*, while *Rest* consisted in the ‘EC’ condition. The rationale at the basis of MDrow index was the well-recognized assumption in literature that drowsiness consists in a vigilance decrease with a parallel reduction of sensorial and cognitive processing (Kamran et al., 2019; Shen et al., 2006). It is assumed that a resting state with closed eyes is the condition representing the greater suppression of cognitive and sensory processes, thus characterized by the highest values of alpha brain rhythms (Mathewson et al., 2011). According to this assumption, the similarity between the Alpha activity during the task of interest and the Alpha activity during a closed eyes resting state condition provides an indication of the drowsy state: the closest to 1 the ratio is (or even higher than 1), the more the individual is drowsy. In this way, the index is positively related to the fatigue level.

#### Electrooculographic assessment

For the collection of EOG data, a minimally invasive approach was also adopted. Specifically, EOG activity was estimated directly from the EEG signal, using the AFz channel to capture the vertical component of ocular movements. This method enabled accurate detection of ocular activity without the need to place electrodes near the eyes, thereby reducing the invasiveness of EOG monitoring while driving. Eyeblink detection was performed using a customized implementation of the Reblinca algorithm (Di Flumeri et al., 2016). From the raw EEG signal, Reblinca algorithm extracted blink events, which were then used to calculate eyeblink rate (EBR), eyeblink duration (EBD), and eyeblink amplitude (EBA).

EBR refers to the number of blink-related artifacts detected within a defined time window, typically expressed in blinks per min, and serves as an indicator of blink frequency. EBD represents the temporal span of each blink artifact, measured in milliseconds, and reflects the duration of eyelid closing and reopening process. EBA quantifies the peak amplitude of the blink-induced artifact on the EEG signal.

#### Photoplethysmographic assessment

For PPG signal it was adopted a non-invasive wearable device, the Empatica E4 wristband (https://www.empatica.com/en-eu/research/e4/), placed on the non-dominant wrist of participants. PPG signal, recorded with a sampling frequency of 64 Hz, was used to estimate heart rate (HR) and heart rate variability parameters in the frequency domain. These consisted in low frequencies (LF), high frequencies (HF), and their ratio, LF/HF (labelled hereinafter HRV). The recorded PPG raw signal was filtered using a 5th-order Butterworth band-pass filter (0.4–4 Hz) to reject the continuous component and the high-frequency interferences, such as that related to movements. A further reason for applying the band-pass filter was to emphasize the “pulse” process of the PPG signal (Goovaerts et al., 1976). These wave patterns related to the pulse, i.e., the beats, were then detected using the Automatic Multiscale-based Peak Detection algorithm (Charlton et al., 2022; Ismail and Karwowski, 2020; Pankaj et al., 2021) and the temporal distance between consecutive beats (inter-beats interval, IBI) was measured to compute the HR values every 60 s. The raw PPG was first segmented into 30-second windows with a 5-second overlap to ensure sufficient temporal resolution while maintaining robustness against transient noise. Within each window, inter-beat intervals were extracted, and artifact detection was applied directly to the IBI time series rather than the raw PPG waveform. Artifact identification relied on standard deviation criteria. In this approach, each IBI was compared against the mean of the detrended IBI series, and values exceeding ±2 standard deviations were flagged as outliers and corrected with local mean, thereby reducing the impact of abrupt signal fluctuations while preserving physiological variability. To further account for spurious trends, the data were linearly de-trended, and smoothing was applied using a moving average filter with a 5-point window. Importantly, these correction procedures were not performed on the PPG signal itself but exclusively on the derived IBI series, given the susceptibility of amplitude measures to movement-related artifacts. The IBI signal was also used to estimated heart rate variability. Specifically, it was analyzed in the frequency domain by computing the Lomb-Scargle periodogram (Ruf, 1999) of the IBI signal. Previous analysis demonstrated that this method is able to provide a more accurate estimation of Power Spectrum Density (PSD) in respect to the Fourier Transform-based methods typically used for HR data (Simonetti et al., 2023). Following best practices present in literature, the PSD of the IBI signal was computed over the Low and High Frequencies (LF: 0.04–0.15 Hz; HF:0.15–0.4 Hz). The ratio between LF and HF values, each of them normalized with respect to the entire spectrum, was then computed as a relevant indicator of heart rate variability (HRV). The HRV analysis was performed using a MATLAB toolbox, the HRVAS MATLAB suite (Ramshur, 2010). When processing the PPG signal, artifacts due to movements were removed interpolating the data between two portions of good quality data.

### Analysis design

In the present study, subjective and neurophysiological fatigue assessment was performed only on the low-demanding driving task (please refer to Figure 2), for two reasons. The first and main reason was that, coherently with RQ1 and literature review, the 45-min-long low-demanding driving allowed the adoption of the most common ToT-driven approach to define periods of low (beginning) and high fatigue (conclusion). The second reason was that the high number of motion artifacts due to the intense driving activity (high speed on a complex circuit) during high-demanding driving would lead to an inaccurate estimation of neurophysiological features, undermining the reliability of the driver’s state assessment.

Also, it has to be considered that, considering the whole 45 min low-demanding driving task, participants were performing different motor activities (i.e., they could be either driving straight or turning left or right). For this reason, the position of the vehicle along the experimental driving path was used to isolate those moments in which participants were driving on the longest straight portion of the path (circle in yellow in Figure 1). The analysis was then performed considering only these portions of the data, where every driver was performing the same identical activity, that is driving straight without the need of controlling the direction, acting on the wheel and on the gearbox (around 1 min duration). This procedure was adopted to minimize the behavioral and cognitive differences in the epochs considered.

The main aim of the present work was to compare two different approaches in investigating drivers’ mental states while driving (RQ1). One approach consisted on the common adoption of ToT to define fatigue levels (low fatigue at the beginning, high fatigue at the end for everyone), while the second approach, i.e., the innovative solution proposed by this work, consisted in using the EEG-derived MDrow index (Di Flumeri et al., 2022) as objective ground-truth to identify and label fatigue levels individually. Consequently, two kinds of segmentation and analysis were performed on the data. In the first segmentation (referred to as “ToT-estimated fatigue”), “Low fatigue” was defined as the first two repetitions of the longest straight road (beginning of the driving task). Conversely, “High fatigue” was defined as the last two repetitions of the longest straight road (end of the driving task). This segmentation represents the assumption, well present in literature, that the lowest level of fatigue is experienced at the beginning of a task while the highest level of fatigue is experienced at the end of a task. In the second segmentation (referred to as “EEG-estimated fatigue”), MDrow was used to detect the individual (i.e., for each participant) time windows in which drivers experienced the highest and the lowest level of fatigue during the low-demanding driving task. A value for each min was computed and the two one-min segments (i.e., two repetitions of the straight road) in which the MDrow showed the highest and the lowest values were labelled as “High fatigue” and “Low fatigue” conditions respectively.

In both cases, the labelled time windows were then used to segment the other neurophysiological parameters to investigate eventual differences due to the intensity of fatigue comparing “Low fatigue” and “High fatigue” conditions. To normalize data, the first 2 min of the low-demanding driving task were taken as baseline. This choice was made to establish a baseline comparable to the investigated task. Using the driving task allowed us to account for brain activity related to motor control (e.g., steering wheel, pedals, etc.). Neurophysiological data used to characterize fatigue (HR, HRV, LF, HF, EBR, EBD, and EBA) were then normalized subtracting the baseline of the corresponding feature. Questionnaire data and the MDrow index were not normalized, since they are directly comparable between participants. To answer RQ2, subjective and physiological responses in simulated and real driving conditions have been then compared. We implemented linear mixed-effects models (LMMs) to analyze each neurophysiological measure and questionnaires separately, with the measure serving as the dependent variable. The models included three fixed factors: Environment (Simulated vs. Real), Fatigue (Low vs. High), and Location (Italy vs. Spain). The Location factor accounted simultaneously for differences in vehicle type, driving route, and other location-specific characteristics. The temporal gap between simulated and real driving sessions was not modeled explicitly, as this information was redundant: all Italian participants performed real driving 6 months after the simulation within a two-day period, whereas Spanish participants completed real driving 5 months after the simulation, also within a two-day period. Participant identity was entered as a random effect, with both random intercepts and random slopes specified, thereby allowing baseline levels and the influence of predictors to vary across individuals. Post hoc power analysis was computed for significant factors and/or interaction of multiple factors. This analysis was computed considering n = 14, and α = 0.05.

Following the LMM analysis, a series of post-hoc t-tests were conducted.

Gaussian distribution of each variable was verified using Shapiro-Wilk test, and parametric or non-parametric post-hoc test was applied accordingly. To account for multiple comparisons, p-values were adjusted using the Holm–Bonferroni method.

The RQ1 working hypothesis is that an EEG-driven approach should enhance the accuracy of the analyses and increase sensitivity to the phenomenon, by enabling a more precise, subject-specific segmentation of the data and thereby reducing the impact of inter-individual variability. If confirmed, we expect the EEG-driven approach to yield larger effect sizes compared to the more conventional time-on-task (ToT-based) approach. Regarding RQ2, no specific working hypothesis was formulated, given the scarcity of empirical evidence supporting differences between simulated and real driving conditions.

## Results

In this section, the results of questionnaires are presented first. After this, two parallel and similar analyses are presented regarding the neurophysiological characterization of fatigue during the driving task, one using the MDrow to define “Low fatigue” and “High Fatigue” conditions (EEG-estimated fatigue), the second analysis assuming the “Low fatigue” condition as the beginning of the driving task, and the “High fatigue” condition at the end of the driving task (ToT-estimated fatigue).

### Subjective assessment

#### Karolinska Sleepiness Scale

**FIGURE 3:**
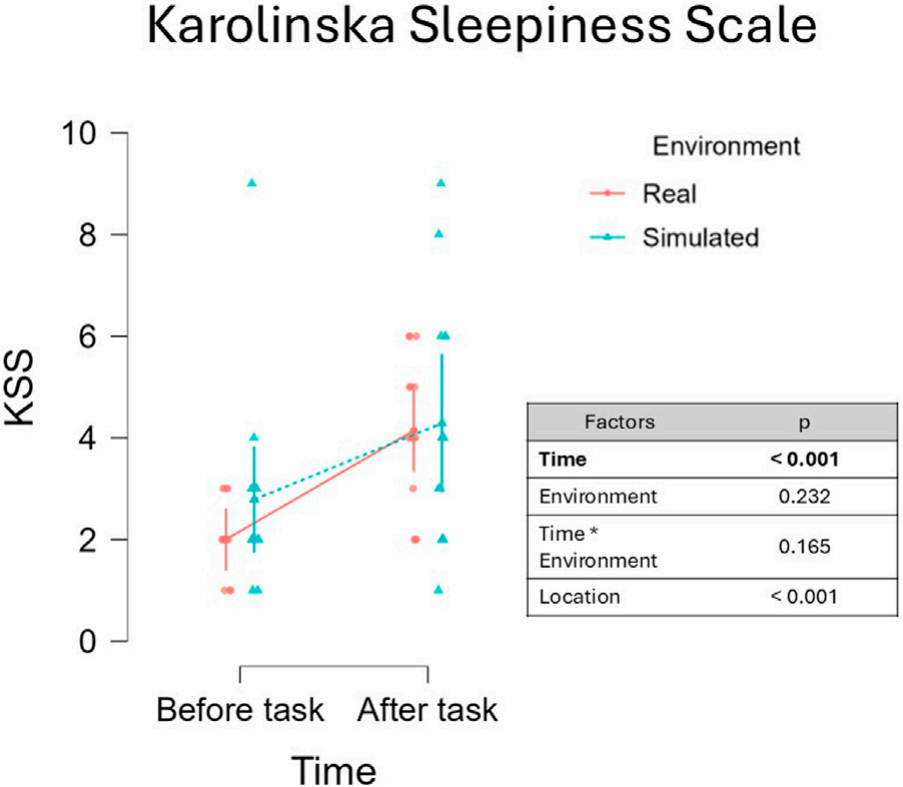
Analysis of KSS scores before and after the driving tasks highlighted a significant increase of fatigue which was consistent in both simulated a real driving (p < 0.001). It is relevant to observe that the median reported KSS score was 4, below the threshold of sleepiness (6, on a scale from 1 to 9). This result demonstrates that the protocol induced a moderate level of fatigue rather than sleepiness.

Statistical analysis revealed a significant main effect of Time on KSS scores, β = −0.896, 95% CI [−1.388 -0.584], F(1, 11.999) = 19.066, p < 0.001 (Figure 3). The effect of Time (t = −4.366, df = 12) yielded a large effect size (Cohen’s f ≈ 1.26), and the estimated power was approximately 98%, indicating strong sensitivity to detect this effect. No effect was observed for the effect Environment, β = −0.250, 95% CI [−0.640 0.140], F(1, 12.704) = 1.572, p = 0.232, as well as the interaction of the two factors, β = −0.177, 95% CI [−0.420 0.066], F(1, 23.80) = 2.053, p = 0.165. Statistical analysis revealed no significant main effect of Location, β = −0.281, 95% CI [−1.081, 0.519], F(1, 12.01) = 0.588, p = 0.458. The effect of Location (t = −0.767, df = 12) yielded a small effect size (Cohen’s f ≈ 0.22), and the estimated power was approximately 11%, indicating low sensitivity to detect this effect.

#### Chalder Fatigue Scale

**FIGURE 4:**
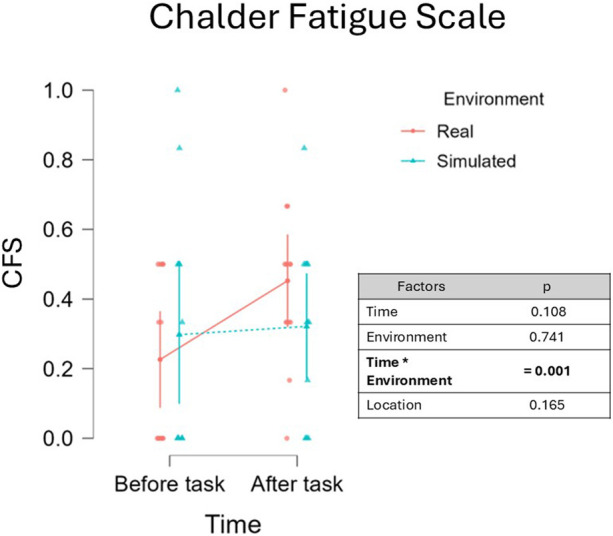
Analysis of CFS scores before and after the driving task revealed a trend toward increased fatigue during the post-task period (p = 0.082). A significant interaction between the fixed effects of Fatigue and Environment was observed (p < 0.001), suggesting that self-perceived fatigue varied between the simulated and real-world conditions. However, the CFS may exhibit limited sensitivity to the onset of fatigue, as the mental symptoms it assesses are not easily triggered by a short (45-min), low-demanding driving task.

Statistical analysis revealed a not significant effect of Time on CFS scores, β = −0.056, 95% CI [−0.119 -0.112], F(1, 12.00) = 3.021, p = 0.108 (Figure 4). No effect was observed for the effect Environment, β = 0.009, 95% CI [−0.042 0.060], F(1, 12.00) = 0.115, p = 0.741, while the analysis of the two factors Environment and Time results in a significant main interaction, β = −0.050, 95% CI [−0.073 -0.026], F(1,12.00) = 16.764, p = 0.001. The effect of the interaction between Task and Time (t = −4.094, df = 12) yielded a very large effect size (Cohen’s f ≈ 1.18), and the estimated power was approximately 97. Post-hoc analysis revealed a significant increase in CFS values after the driving task in the Real environment, T-Stat (39) = 2.826, p = 0.047, Cohen’s d = −0.823. No significant differences emerged between the remaining comparisons (all p > 0). In addition, a significant effect of the fixed effect factor Location was observed, β = −0.184, 95% CI [−0.245 -0.123], F(1, 12.00) = 35.343, p < 0.001. The effect of Location (t = −5.945, df = 12) yielded a very large effect size (Cohen’s f ≈ 1.72), and the estimated power was approximately 100%.

### Different time windows–EEG-estimated fatigue vs. ToT-estimated fatigue

**FIGURE 5:**
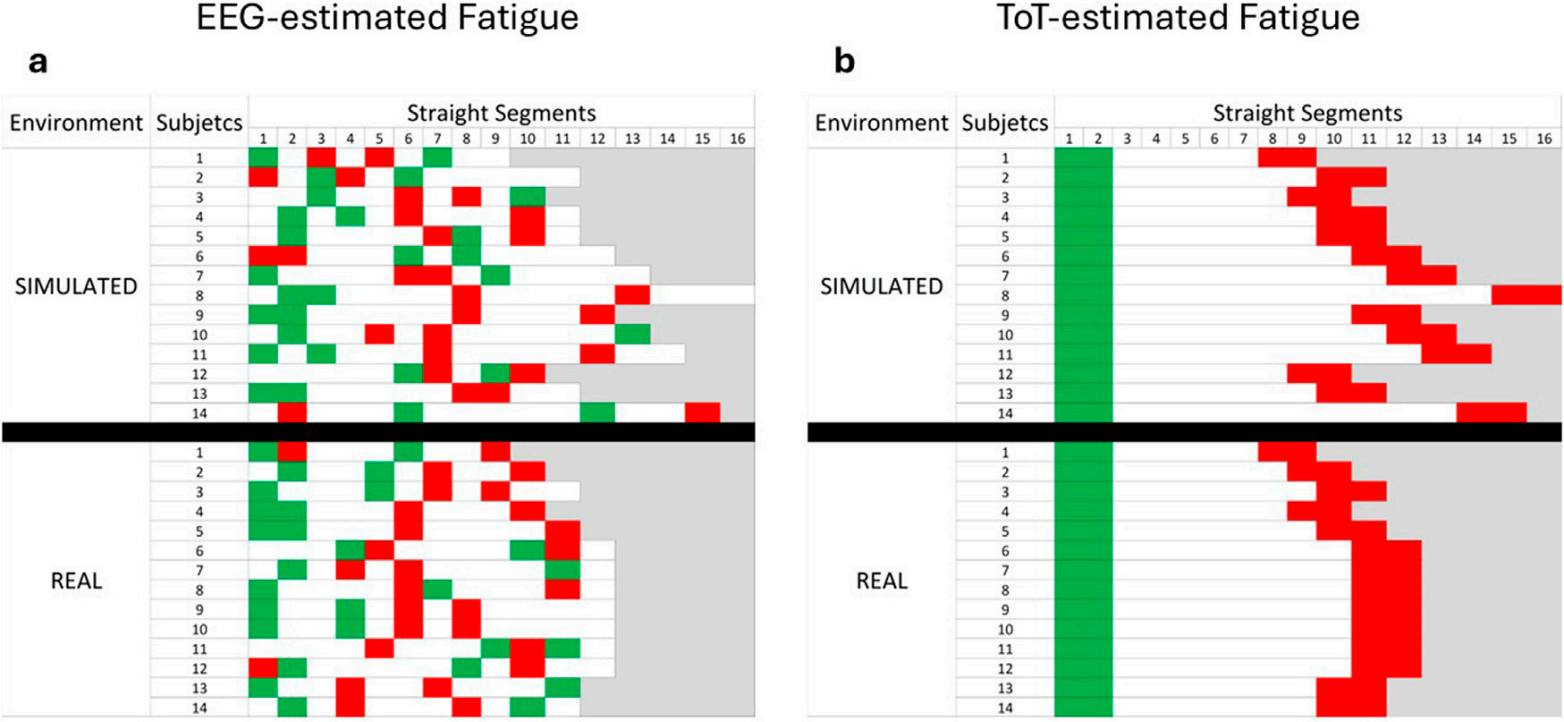
Representation of low and high fatigue periods defined with both the EEG-driven approach **(a)** and the ToT-driven approach **(b)**. In b, all the participants are supposed to experience the lowest and the highest level of fatigue synchronously. In a, EEG-driven segmentation revealed a wide interindividual variability in the timing of both lowest and highest fatigue periods.

Figure 5 illustrates the segmentation outcomes based on the EEG-driven (on the left) and ToT-driven (on the right) approaches for both simulated and real driving environments. In the ToT-driven approach (Figure 5b), low and high fatigue conditions were defined *a priori* as the initial and final parts of the driving task, respectively.

As a result, the segmentation is identical across participants, with green and red segments consistently marking the early (low fatigue) and late (high fatigue) portions of the task. Conversely, the EEG-driven approach (Figure 5a) relied on individual EEG data, using the MDrow index to identify the time segments corresponding to minimum (low fatigue, green) and maximum (high fatigue, red) fatigue levels for each participant. This method naturally resulted in a high degree of inter-individual variability in the segmentation timing. While low fatigue conditions tend to be concentrated toward the beginning of the task (left side of the figure) and high fatigue conditions toward the end (right side), the exact location and distribution of these segments differ markedly between participants, reflecting the personalized nature of EEG-based fatigue detection.

### Neurophysiological assessment of fatigue onset

#### MDrow

**FIGURE 6:**
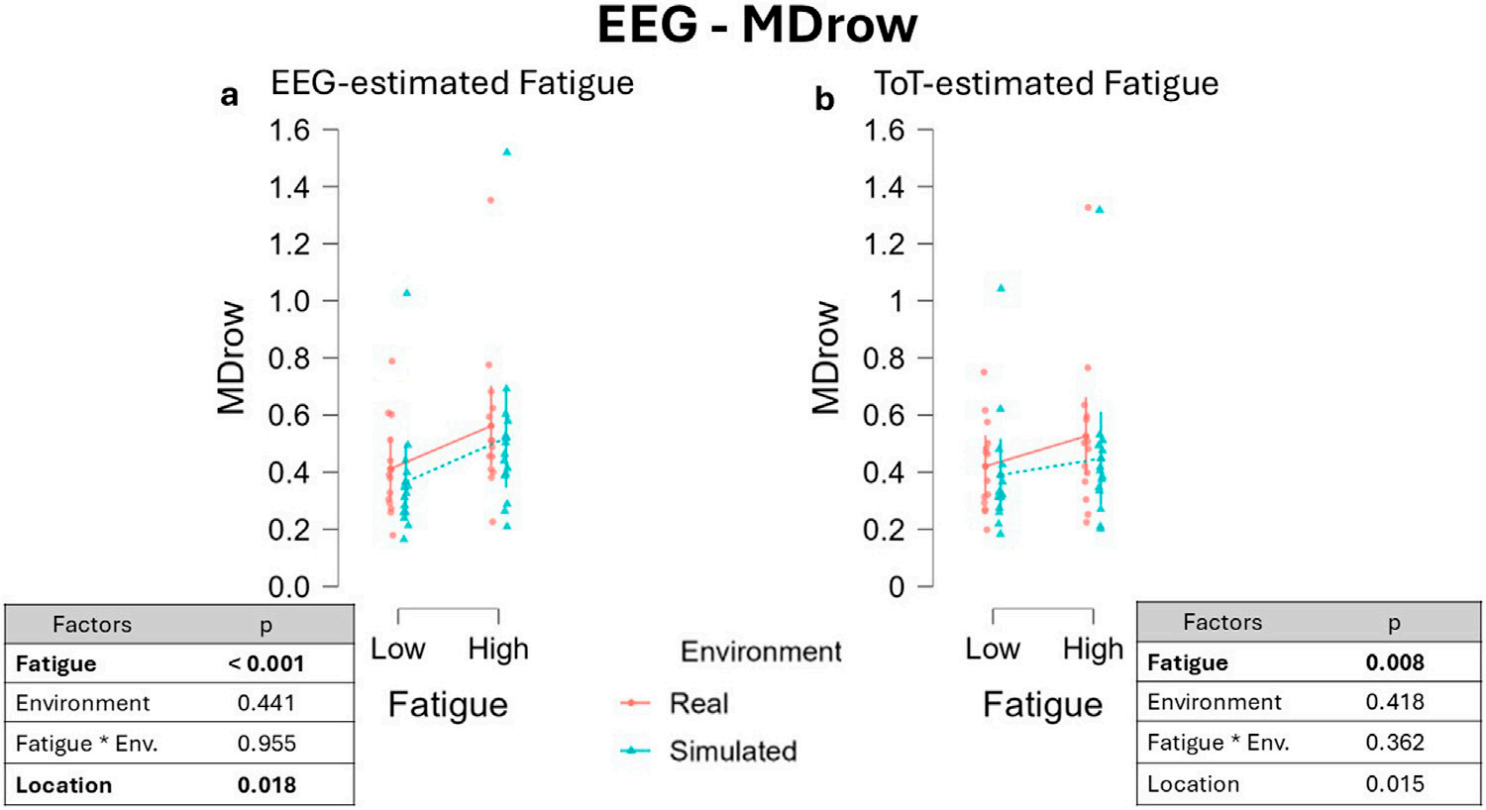
MDrow variations across fatigue conditions using two different segmentation approaches: the EEG-driven approach **(a)** and the ToT-driven approach **(b)**. No significant differences emerged from the interaction between the factors Fatigue and Environment, indicating that the intensity of the experienced fatigue was comparable across the two environments.

Of course, running the LMM following the EEG-estimated fatigue segmentation, a significant main effect of the fixed effect Fatigue, β = −0.081, 95% CI [−0.106 −0.055], F(1,16.22) = 37.387, p < 0.001 is found (Figure 6a), with higher MDrow during the ‘High fatigue’ condition according to the research hypothesis. The estimated power to detect an effect of Fatigue was approximately 99% (with t = −6.115, df = 13), which corresponds to a big effect size (Cohen’s f = 1.70). No significant effect of Environment was observed, β = 0.028, 95% CI [−0.040 0.096] F(1,13) = 0.631, p = 0.441. No effect was observed for the interaction between the two fixed effects Fatigue and Environment, β = −6.106 × 10^−4^, 95% CI [−6.642 × 10^−4^ 6.6 × 10^−4^] F(1, 26.00) = 0.003, p = 0.955. A significant effect of the main fixed effect Location was observed, β = −0.113, 95% CI [-0.198 -0.042], F(1,12.004) = 7.293, p = 0.018. The Location effect (t = −2.701, df = 13) corresponded to a large effect size (Cohen’s *f* = 0.75). The estimated statistical power for detecting this effect was approximately 90%.

Considering the ToT -driven segmentation, LMM results revealed a significant main effect of the fixed effect Fatigue, β = −0.044, 95% CI [−0.073 -0.015], F(1, 16.00) = 6.073, p = 0.008. The effect of Fatigue (t = −3.017, df = 16.548) corresponded to a medium-to-large effect size (Cohen’s *f* ≈ 0.74), with an estimated power of approximately 88%.

No effect was observed either on fixed effect Environment, β = 0.029, 95% CI [−0.038 0.096], F(1, 13.00) = 0.704, p = 0.418, nor its interaction with fixed effect Fatigue, β = −0.016, 95% CI [−0.041 0.009], F(1, 26.00) = 0.802, p = 0.362, Figure 6b). A significant effect of the main fixed effect Location was observed, β = −0.113, 95% CI [−0.191 -0.035] F(1,12) = 7.963, p = 0.015. The effect of Location (t = −2.822, df = 12) yielded a large effect size (Cohen’s f ≈ 0.82), and the estimated power was approximately 93%, indicating strong sensitivity to detect this effect.

#### EOG

Analysis of EOG data segmented using MDrow (EEG-estimated fatigue) revealed a different impact across the three blink-related measures.

**FIGURE 7:**
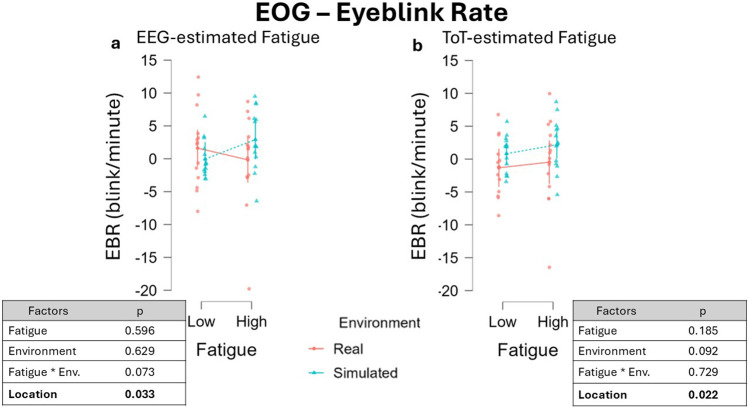
EBR variations across fatigue conditions using two different segmentation approaches. **(a)** Values obtained defining fatigued periods through the EEG-driven approach highlighted no significant changes considering the single factors Fatigue and Environment. A marginal effect of the interaction between the fixed effects Fatigue and Environments was observed (p = 0.062). **(b)** Conversely, defining fatigue with the ToT-driven approach resulted in no significant changes in EBR. Data were normalized by subtracting baseline value.

LMM run on EBR data resulted in an absence of significant effect for both the fixed effects Fatigue, β = −0.377, 95% CI [−1.747 0.993], F(1, 19.38) = 0.291, p = 0.596, and Environment, β = −0.329, 95% CI [−1.646 0.988], F(1, 27.87) = 0.239, p = 0.629 (Figure 7a). The interaction between the two factors revealed a tendency to a significant effect which did not reach the significant level, β = 1.190, 95% CI [−0.074 2.454], F(1, 36.00) = 3.406, p = 0.073. A significant effect was observed for the factor Location, β = −1.787, 95% CI [−3.278 −0.295], F(1, 14.83) = 5.507, p = 0.033. The effect of Location on EBR (t = −2.347, df = 15) yielded an effect size of Cohen’s f ≈ 0.61, and the estimated power was approximately 58%, indicating weak sensitivity to detect this effect.

Following the ToT-driven segmentation, no significance was found for both factors Fatigue, β = −0.546, 95% CI [−1.353 0.261], F(1, 12.00) = 1.753, p = 0.210, and Environment, β = −1.207, 95% CI [−2.199 0.145], F(1, 12.01) = 6.950, p = 0.112. No effect was found also for the interaction of the two fixed factors Fatigue and Environment, β = 0.017, 95% CI [−1.155 1.189], F(1, 12.00) = 0.003, p = 0.959 (Figure 7b), while a significant effect of the fixed effect factor Location was observed, β = −1.913, 95% CI [−3.334 −0.492], F(1, 12) = 6.950, p = 0.022. The impact of Location factor (t = −2.636, df = 12) yielded an effect size of Cohen’s f ≈ 0.761, and the estimated power was approximately 68%, indicating weak sensitivity to detect this effect.

**FIGURE 8:**
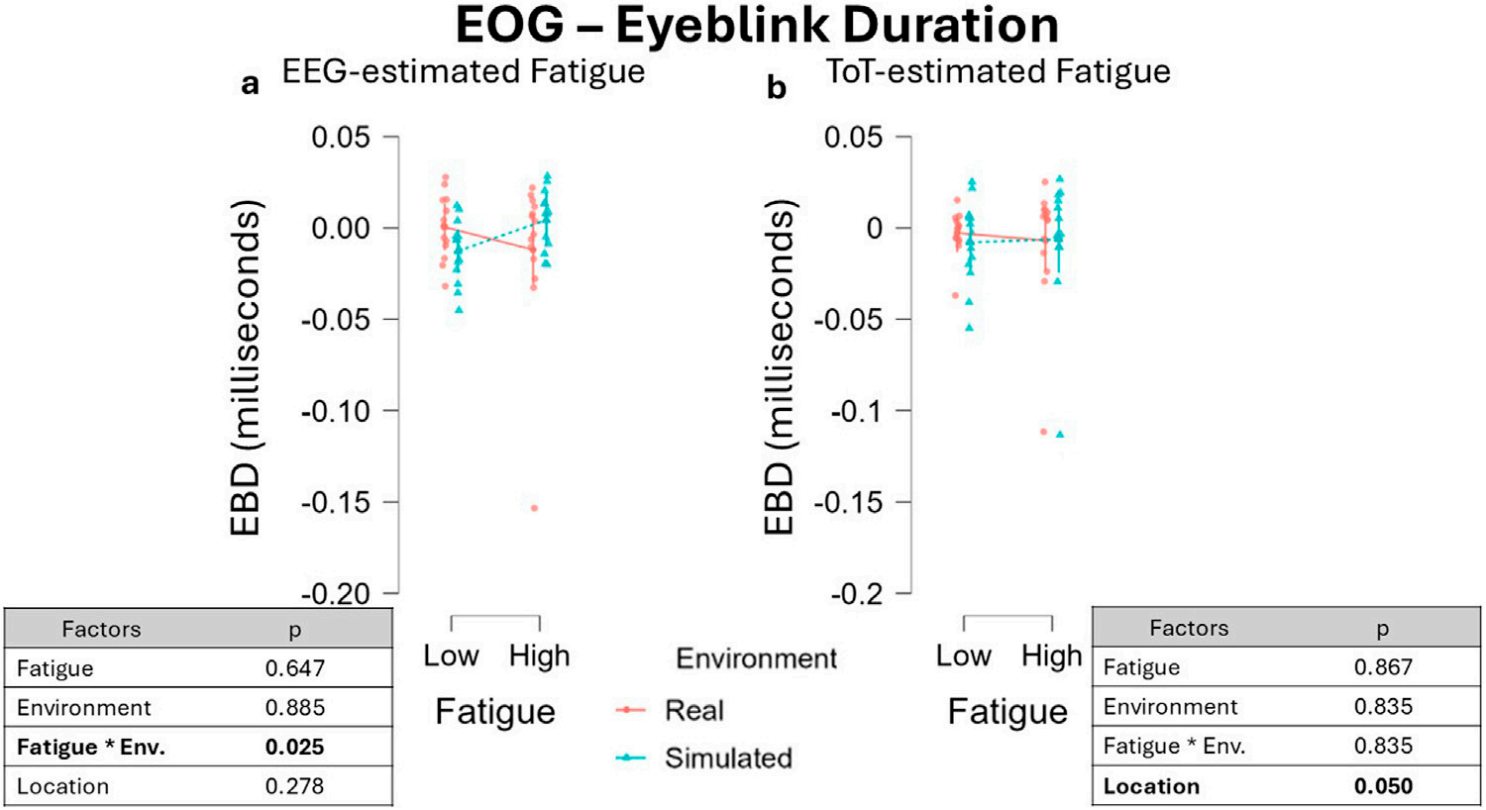
EBD variations across fatigue conditions using two different segmentation approaches. **(a)** Values obtained defining fatigued periods through the EEG-driven approach highlighted a significant interaction between the fixed effects Fatigue and Environments (p = 0.013). **(b)** Conversely, defining fatigue with the ToT-driven approach resulted in no significant changes in EBD. Data were normalized by subtracting baseline value.

Using the EEG-driven approach, a similar dynamic was observed in EBD feature, with the difference that the interaction between the two fixed effects Fatigue and Environment was found to reach the significance threshold, β = 0.007, 95% CI [0.001 0.012], F (1, 36.00) = 5.446, p = 0.025 (Figure 8a). The effect of the interaction between the factors Fatigue and Environment on EBD (t = −2.334, df = 36) yielded an effect size of Cohen’s f ≈ 0.37, and the estimated power was approximately 43%, indicating weak sensitivity to detect this effect. Following LMM analysis, post-hoc t-tests were conducted for all pairwise comparisons. However, none of these comparisons reached statistical significance. No effect was found for the single factor Fatigue, β = −0.002, 95% CI [−0.009 -0.004], F(1, 14.60) = 0.219, p = 0.647, as well as for the factor Environment, β = −4.708 × 10^−4^, 95% CI [−4,713 × 10^−4^ −4.702 × 10^−4^], F(1, 21.07) = 0.021, p = 0.885. No effect was found for the fixed factor Location, β = -0.005, 95% CI [-0.012 0.002], F(1, 12.72) = 1.283, p = 0.278.

Considering the ToT segmentation, no effect nor tendency was observed in EBD data for both the factors Fatigue, β = 4.884 × 10^−4^, 95% CI [4.878 × 10^−4^ 4.889 × 10^−4^], F(1, 13.05) = 0.029, p = 0.867, and Environment, β = 9.252 × 10^−4^, 95% CI [9.244 × 10^−4^ 9.260 × 10^−4^], F(1, 12.02) = 0.045, p = 0.835, as well as for their interaction, β = 0.001, 95% CI [−0.002 0.005], F(1, 24.00) = 0.458, p = 0.835 (Figure 8b). A significant effect was found also for the fixed effect factor Location, β = −0.010, 95% CI [-0.017 -0.002], F(1, 12.00) = 4.752, p = 0.050. The impact of Location factor on EBD (t = −2.180, df = 12) yielded an effect size of Cohen’s f ≈ 0.627, and the estimated power was approximately 52%, indicating weak sensitivity to detect this effect.

**FIGURE 9:**
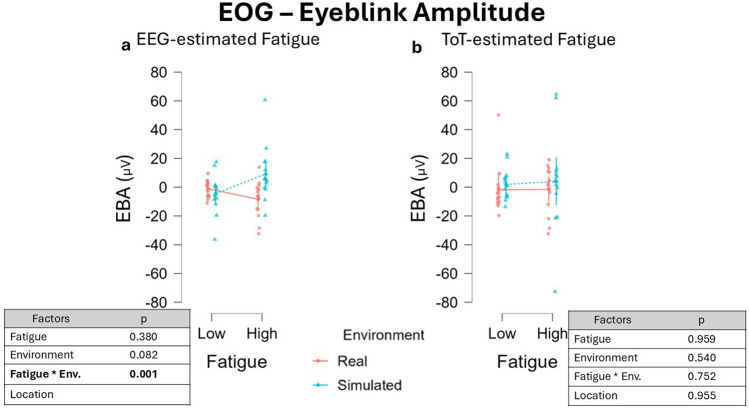
EBA variations across fatigue conditions using two different segmentation approaches. **(a)** Values obtained defining fatigued periods through the EEG-driven approach highlighted a marginal increase of blink amplitude in the simulated environment compared to the real one (Environment, p = 0.066). In addition, a significant interaction between the fixed effects Fatigue and Environments was observed (p = 0.013). **(b)** Conversely, defining fatigue with the ToT-driven approach resulted in no significant changes in EBA. Data were normalized by subtracting baseline value.

LMM analysis on EBA data segmented with EEG resulted in a tendency to a significant effect of the fixed effect Environment, β = −3.514, 95% CI [−7,130 0.102], F (1, 12.38) = 3.579, p = 0.082, which was observed to be higher in simulated driving (Figure 9a). No effect was observed for the factor Fatigue, β = −1.912, 95% CI [−5.528 1.704], F(1, 13.25) = 0.824, p = 0.380. Conversely, a significant interaction between Environment and Fatigue was observed, β = 5.567, 95% CI [2.576 8,557], F(1, 24.00) = 13.314, p = 0.001. The effect of the interaction between the factors Fatigue and Environment on EBA (t = 0.863, df = 14) yielded an effect size of Cohen’s f ≈ 0.227, and the estimated power was approximately 18%, indicating weak sensitivity to detect this effect. Post-hoc analysis revealed no significant differences in terms of EBA between all the comparisons investigated. However, EBA during ‘High’ fatigue periods in Simulated environments showed a tendency to an increase compared to both low fatigue periods in Simulated driving, T-Stat = 2.465, p = 0.091, and also compared to “High” fatigue periods in Real driving, T-Stat = 2.610, p = 0.077. No effect was observed for the fixed effect factor Location, β = 0.918, 95% CI [−2.698 4.534], F(1, 24.00) = 12.314, p = 0.001. Running the analysis on EBA data segmented with a ToT-driven approach, no effect for any of the factors considered was observed (Figure 9b). Both the factors Fatigue, β = −0.167, 95% CI [-6.417 6.083], F(1, 12.33) = 0.003, p = 0.959, and Environment, β = −2.301, 95% CI [−9.455 4.853], F(1, 12.10) = 0.397, p = 0.540, did not impact significantly on EBA. The interaction between the two fixed effects was also investigated and no significant effect was observed, β = 0.532, 95% CI [−2.723 3.787], F(1, 24.00) = 0.103, p = 0.752. No effect was found also for the fixed effect factor Location, β = −0.161, 95% CI [−5.639 5.317], F(1, 12.20) = 0.003, p = 0.955.

#### PPG

**FIGURE 10:**
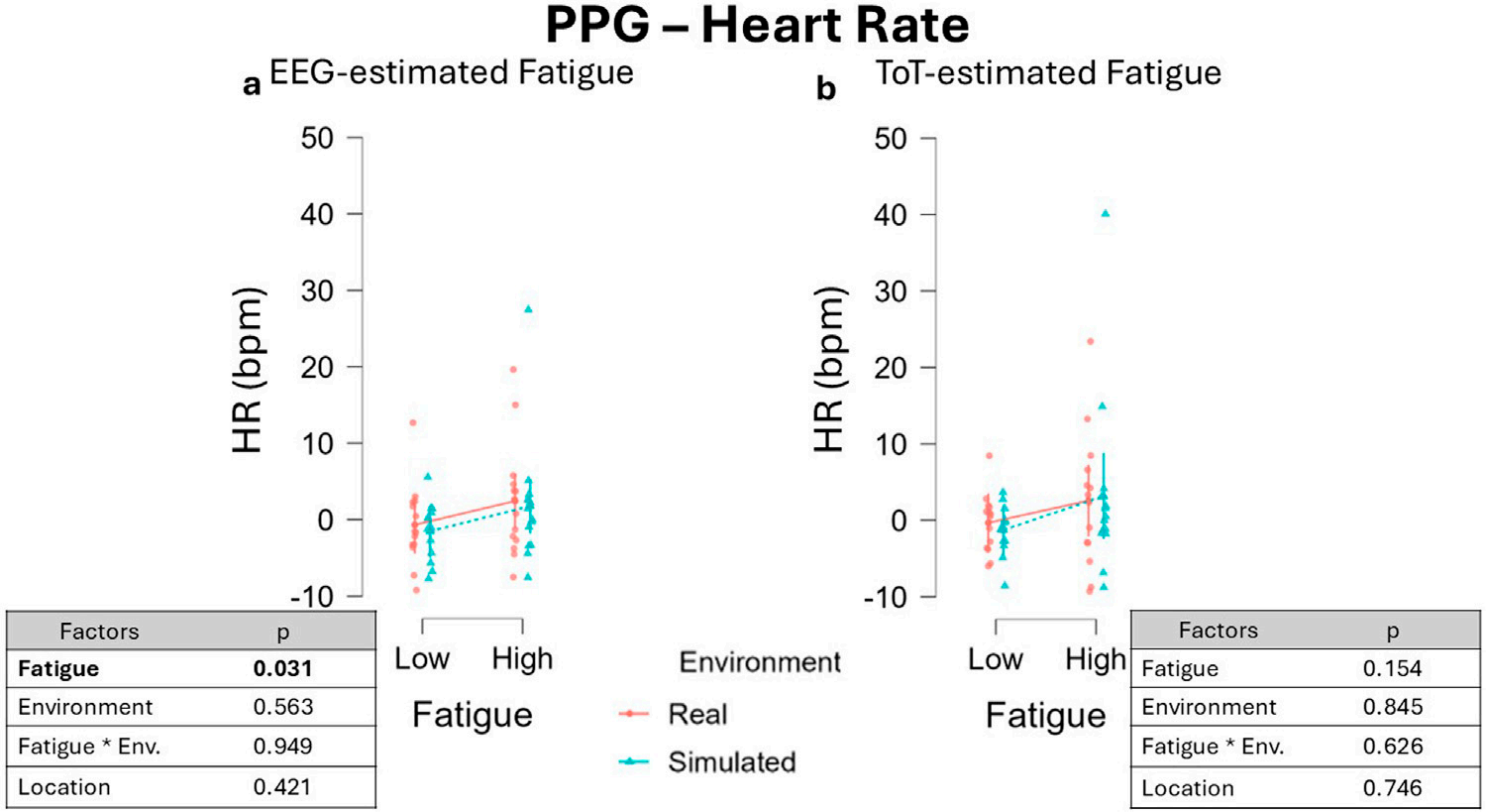
HR variations across fatigue conditions using two different segmentation approaches. **(a)** When fatigue periods were defined using the EEG-driven approach, HR was significantly higher during high fatigue compared to low fatigue across both simulated and real driving environments (p = 0.038). **(b)** No significant effect of fatigue or environment on HR was observed when using the ToT-driven approach. Data were normalized by subtracting baseline value.

The EEG-driven approach highlighted an increase in HR during fatigued periods, as highlighted by the significant main effect of the factor Fatigue, β = −1.593, 95% CI [−2.986 −0.199], F(1, 35.71) = 5.025, p = 0.031 (Figure 10a). The effect of Fatigue (t = −2.242, df = 36) yielded an effect size of Cohen’s f ≈ 0.37, and the estimated power was approximately 55%, indicating weak sensitivity to detect this effect. On the contrary, driving in the Simulated or Real environment did not impact on HR while driving, Environment, β = 0.656, 95% CI [−1.507 2.820], F(1, 13.05) = 0.353, p = 0.563. The interaction between the two factors Fatigue and Environment was also investigated, resulting in a not significant effect, β = 0.173, 95% CI [−1.202 1.549], F(1, 39.00) = 0.004, p = 0.949. No significant main effect of Location on HR was observed, β = −0.572, 95% CI [–1.950, 0.806], F(1, 38.56) = 0.662, p = 0.421.

Taking into account data segmented using ToT-driven approach, it was observed no significant effect of both Fatigue, β = 1.440, 95% CI [−0.453 3.333], F(1, 17.08) = 2.220, p = 0.154, and Environment, β = 0.215, 95% CI [-1.197 2.347], F(1, 16.04) = 0.039, p = 0.845, as well as no effect was observed for their interaction, β = −0.283, 95% CI [1.125 2.706], F(1, 30.12) = 0.242, p = 0.626 (Figure 10b). Statistical analysis revealed no significant main effect of Location on [outcome measure], β = 0.300, 95% CI [–1.491, 2.091], F(1, 17.11) = 0.108, p = 0.746.

**FIGURE 11:**
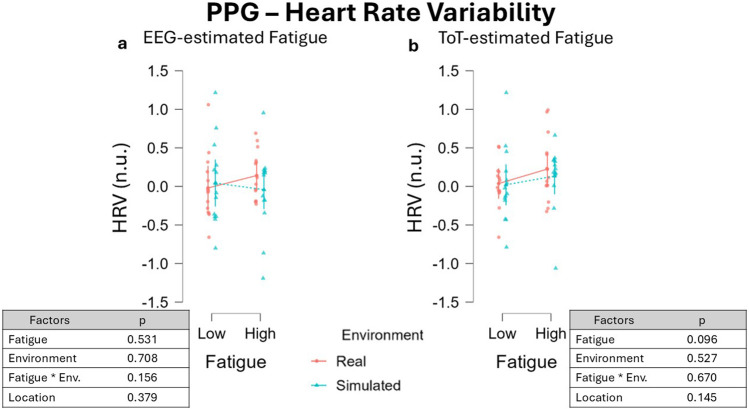
HRV variations across fatigue conditions using two different segmentation approaches: the EEG-driven approach **(a)** and the ToT-driven approach **(b)**. Both approaches resulted in no significant impact of Fatigue as well as Environment on HRV. Data were normalized by subtracting baseline value.

LMM run on HRV data segmented using the EEG-driven approach resulted in an absence of significant effect of the two factors Fatigue, β = −0.032, 95% CI [−0.128 0.064], F(1, 13.59) = 0.413, p = 0.531, and Environment, β = 0.028, 95% CI [-0.117 0.173], F(1, 12.02) = 0.147, p = 0.708 (Figure 11a), as well as of their interaction, β = −0.054, 95% CI [−0.126 0.018], F(1, 24) = 2.142, p = 0.156 (Figure 11a). No effect was observed for the fixed effect Location, β = 0.071, 95% CI [−0.081 0.223], F(1, 12.04) = 0.835, p = 0.379.

Analogous results were observed for the ToT-driven approach, where the two factors Fatigue, β = −0.084, 95% CI [−0.185 ],F(1, 15.98) = 3.139, p = 0.096, and Environment, β = 0.034, 95% CI [-0.067 0.136], F(1, 13.50) = 0.421, p = 0.527, did not impact on HRV while driving. Also the interaction between the two main effects was not significant, β = −0.018, 95% CI [−0.102 0.066], F(1, 24.00) = 0.186, p = 0.670 (Figure 11b), as well as the fixed effect Location, β = 0.110, 95% CI [−0.029 0.249], F(1, 12.05) = 2.430, p = 0.145.

**FIGURE 12:**
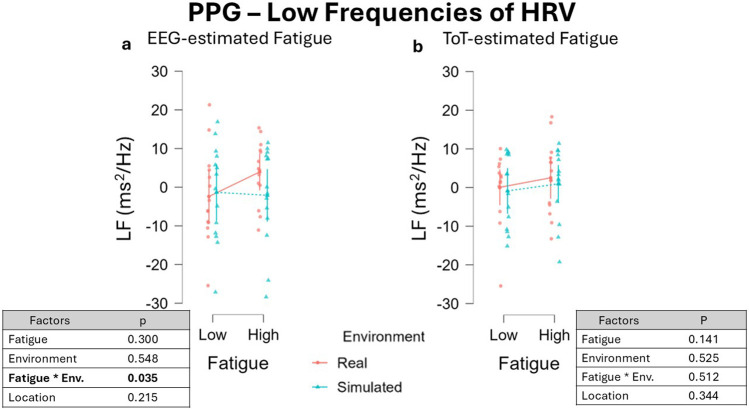
LF variations across fatigue conditions using two different segmentation approaches: the EEG-driven approach **(a)** and the ToT-driven approach **(b)**. Both approaches resulted in no significant impact of Fatigue as well as Environment on LF. Data were normalized by subtracting baseline value.

The single components of HRV were also investigated, LF and HF. Regarding LF, adopting the EEG-driven approach it was observed a significant interaction between the factors Fatigue and Environment, β = −0.176, 95% CI [−0.332 −0.020], F(1, 35.65) = 4.816, p = 0.035 (Figure 12a). The effect of the interaction between the fixed effect factors Fatigue and Environment (t = −1.463, df = 24) yielded an effect size of Cohen’s f ≈ 0.299, and the estimated power was approximately 27%, indicating weak sensitivity to detect this effect. Post-hoc t-test revealed no significant difference between all the comparisons investigated. Considering the single factors, no effect was observed for Fatigue, β = −0.096, 95% CI [−0.272 0.080], F(1, 17.17) = 1.142, p = 0.300, Environment, β = 0.068, 95% CI [-0.147 0.283], F(1, 12.26) = 0.381, p = 0.548. as well as for Location, β = −0.101, 95% CI [−0.258 0.056], F(1, 35.65) = 1.595, p = 0.215.

Adopting the alternative approach, the ToT-driven segmentation, no effect was observed for both Fatigue, β = −0.134, 95% CI [−0.306 0.038], F(1, 22.59) = 2.323, p = 0.141, and Environment, β = 0.054, 95% CI [-1.612 1.520], F(1, 35.84) = 0.413, p = 0.525, as well as no significant effect was observed for the interaction of the two factors, β = 0.066, 95% CI [-0.101 0.224], F(1, 36.00) = 0.398, p = 0.512 (Figure 12b). No effect was observed for the fixed effect Location, β = 0.085, 95% CI [-0.087 0.257], F(1, 24.67) = 0.931, p = 0.344.

**FIGURE 13:**
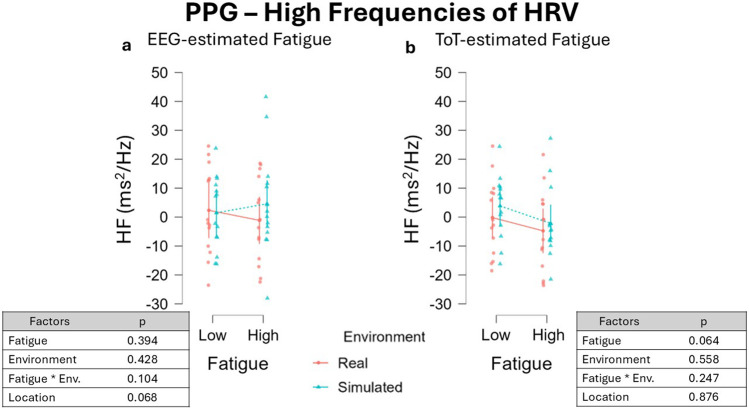
HF variations across fatigue conditions using two different segmentation approaches: the EEG-driven approach **(a)** and the ToT-driven approach **(b)**. Both the approaches resulted in no significant impact of Fatigue as well as Environment on HF. Data were normalized by subtracting baseline value.

Analysis run on HF data segmented with the EEG-driven approach showed no impact of both Fatigue, β = 0.085, 95% CI [−0.107 0.277], F(1, 26.79) = 0.749, p = 0.394, and Environment type, β = −0.075, 95% CI [−0.259 0.109], F(1, 33.09) = 0.643, p = 0.428, as well as no effect was observed considering their interaction, β = 0.156, 95% CI [-0.028 0.340], F(1, 36.00) = 2.780, p = 0.104 (Figure 13a). No significant effect was observed for the fixed effect Location, β = 0.232, 95% CI [0.003 0.461], F(1, 13.41) = 3.947, p = 0.068. Similarly, the ToT-approach in segmenting HF data showed analogous results: no significant effect of Fatigue, β = 0.191, 95% CI [-0.007 0.389], F(1, 48.00) = 1.893, p = 0.064, and Environment, β = −0.060, 95% CI [−0.258 0.138], F(1, 48.00) = 0.349, p = 0.558, nor of their interaction, β = −0.118, 95% CI [−0.316 0.078], F(1, 48) = 0.1.371, p = 0.247 (Figure 13b). No effect was observed for the fixed effect Location, β = 0.016, 95% CI [−0.182 0.214], F(1, 48.00) = 0.024, p = 0.876.

## Discussion

The ability to detect fatigued states derives from decades of academic and industrial research. Researchers have long worked to characterize the physiological (Bundele and Banerjee, 2009; Nguyen et al., 2017; Fujiwara et al., 2019; Alaimo et al., 2020; Arefnezhad et al., 2022) and behavioral (Brandt et al., 2004; Fairclough and Graham, 1999; Ghourabi et al., 2020) markers of fatigue and drowsiness. To induce fatigue, users are often required to perform a task over an extended period. Indeed, several findings in literature support that performing a long-lasting task resulted in a fatigued state, a concept known as Time-on-Task (ToT) effect. According to this framework, fatigue is assumed to increase with task duration. Even if some evidence challenges the universal validity of the ToT effect, the final part of a prolonged driving or cognitive task is typically interpreted as a fatigued state (Ackerman and Kanfer, 2009; Lim et al., 2010; Hopstaken et al., 2015; Behrens et al., 2023). This issue is made worse by two key limitations: (i) fatigue is a spontaneous phenomenon that cannot be directly modulated through task parameters (unlike, for example, mental workload, which can be manipulated by adjusting task difficulty); and (ii) there is no established ground-truth measure that allows for continuous monitoring and quantification of an individual’s fatigue level.

In this context, the primary aim of the present manuscript was to compare the results obtained in assessing mental fatigue onset on drivers with the common ToT-based approach, towards an innovative physiology-driven approach based on the use of EEG-derived parameters (RQ1). To address this, a multi-steps experimental protocol requiring to drive in different conditions was conducted, and the 45-min-long low demanding driving task was analyzed. The first step consisted in verifying whether the experimental protocol induced fatigue. Subjective reports, in the form of questionnaires, were used to collect drivers’ perception of the fatigued state. Two questionnaires were provided, KSS and CFS. As confirmed by subjective reports of sleepiness, participants perceived an increase in fatigue during the low-demanding driving task, although no significant increase of CFS was observed. The values of KSS scored after the driving task were found to be significantly higher than those scored before the task (Figure 3), while values of CFS questionnaires increased, but only marginally (Figure 4). In respect to the CFS (Cella and Chalder, 2010), the marginal increase may be attributed to the nature of the CFS questionnaire, which targets symptoms of fatigue that typically emerge in chronic or prolonged conditions and which are not likely to develop in a 45-min-long low-demanding driving task. Even though only the mental fatigue subscale was considered in this study, CFS questionnaire may be more suitable for studying longitudinal or chronic fatigue, which is more likely to induce the insurgence of the symptoms checked by the questionnaire, such as, for example, the loss of motivation, or difficulty in concentrating. In addition, statistical analysis revealed a significant effect of Location on CFS scores, indicating that self-reported fatigue was influenced by the site at which the experiments were conducted (Italy vs. Spain). Several contextual differences between the two settings may account for this effect, including the experimenters involved, the type of vehicle employed (a van in Italy vs. a truck in Spain), and task-related characteristics such as variations in the driving route (e.g., the presence of curves). However, despite the overall difference in CFS scores between the two locations, no significant interactions were observed between Location and the other fixed-effect factors. This suggests that, although CFS values differed across experimental sites, the pre- to post-task changes in fatigue were consistent and generalizable and not driven by the specific Location. Regarding KSS, it was developed and validated as a questionnaire aimed at investigating drowsiness. Although conceptually distinct, mental fatigue and drowsiness are often viewed as varying intensities along the same continuum from alertness to sleep (Kamran et al., 2019). Due to their overlap and the limited sensitivity of subjective measures, both questionnaires were administered to better capture these states. According to this view, it is interesting to note that even if significantly increased, KSS values after the driving task scored a median of 4, which is below the threshold for sleepiness (equal to 6, on scale from 1 to 9), but higher than the median value of 2 (alertness) scored before the driving task. This consideration supports the idea that what was experienced during the 45-min-long driving task was fatigue rather than sleepiness. Once it was confirmed that the experimental protocol effectively induced fatigue as perceived by participants, it became appropriate to proceed with the comparison between the EEG-driven and the ToT-driven approaches. Indeed, to address RQ1 data collected during a 45-min-long task were labelled as low and high fatigue using both an EEG-driven approach, where the MDrow index (Di Flumeri et al., 2022) was used to detect the lowest and highest fatigue experienced, in parallel with a ToT-driven approach, where the initial and the final segments of the task were considered as the lowest and highest fatigue periods respectively. As illustrated in Figure 5, the EEG-driven and ToT-driven approaches led to different segmentations of the driving task. This divergence highlighted how the choice of fatigue assessment method can substantially influence the observation of fatigue-related changes over time. Looking at the segmentation, it appears evident that the EEG-driven approach presents high variability in the time windows labelled as low and high fatigue for different drivers. The observed high variability might be interpreted as the fact that the EEG-driven approach captured the interindividual variability, revealed by the marked differences between low and high fatigue periods of different users. In contrast, the ToT-driven approach assumes a uniform fatigue progression across all individuals, assigning the same temporal segments as low and high fatigue for every participant, and so disregarding individual differences in terms of fatigue perception and progression over time. Both the EEG-driven and ToT-driven approaches revealed a significant increase in MDrow values during high fatigue compared to low fatigue, in both simulated and real-world settings. However, the EEG-driven approach yielded a stronger statistical significance (p < 0.001) than the ToT-driven approach (p = 0.025) (Figure 6). These findings indicate that brain correlates of fatigue increased in both cases, but segmenting the data based on EEG allowed for a more pronounced distinction between low and high fatigue periods. On one hand, the result on the EEG-driven approach was an implicit consequence of the method: since the MDrow index has been used to segment data into the two conditions (Low and High fatigue), then its comparison is statistically relevant. On the other hand, the analysis of the ToT-driven approach: (i) confirmed the reliability of the MDrow index, that was significantly higher in the end of the experiments; but (ii) also demonstrated the less sensitivity of this approach in identifying the individual periods of highest fatigue. It should be noted that a significant effect of Location was observed on MDrow, for both the EEG-driven and the ToT-driven approaches. Importantly, no significant interactions emerged between Location and the other fixed-effect factors, supporting the generalizability of the results across the two methods, regardless of the site in which the experiments were conducted. The true added value of the innovative approach proposed in this study, however, emerges in the analysis of the additional physiological parameters considered. In fact, it was observed that, by adopting the ToT-driven approach, fatigue onset did not impact on the physiological response of drivers, i.e., absence of significant differences between low and high fatigue for all the features extracted from ocular (EBR, EBD, and EBA) and cardiac (HR, HRV, LF, and HF) activity. None of the factors considered, Fatigue and Environment, as well as their interaction, were found to impact physiological activity. In contrast, by adopting the EEG-driven approach, it was possible to recognize the effect of fatigue onset on physiological response of drivers. Ocular activity showed a different response to fatigue onset depending on whether the driving task was performed in a simulated or real environment. Results of LMM analysis revealed a significant interaction of the factors Fatigue and Environment on eyeblink duration (EBD, Figure 8a) and amplitude (EBA, Figure 9a). However, *post hoc* pairwise comparisons did not result to be significant for either ocular metric. Although *post hoc* pairwise comparisons did not reach statistical significance for either ocular metric, an opposite trend in the impact of fatigue onset was observed across all metrics. As shown in Figures 8a, 9a, during simulated driving, all metrics tended to increase in high fatigue periods compared to low fatigue periods. Conversely, in real driving, the same metrics showed a decreasing trend with fatigue onset. This observation suggests that driving in a simulated or real environment may induce a different response on ocular metrics. This could be induced by several factors. Performing visual task using a screen can induce an alteration of eye moisture (Bafna and Hansen, 2021) which can in turn affects the eyeblink behavior. Indeed, one of the functions of eyeblinks is indeed to regulate the balance of eye moisture levels (Portello et al., 2013). Screens adopted for the simulated task might have caused a change in the moisture levels of the eyes which in turn induced blinking strategies to recover the homeostasis level (Eckstein et al., 2017). Alternatively, the differential response may be attributed to the cognitive and physiological effect of experiencing fatigue while driving in a simulated environment compared to experiencing fatigue driving a real vehicle on the road. Regarding heart activity in response to fatigue onset, measures of heart rate variability, HRV, LF, and HF, were not found to be impacted both by fatigue onset and the driving environment. Changes in HRV-related measures have been widely adopted to characterize fatigue (O’Keeffe et al., 2020; Wilson et al., 2007). An increase in LF has been positively linked to an activation of the sympathetic branch of the autonomous nervous system, which is triggered when users’ arousal or stress increases (Pham et al., 2021). Conversely, literature suggests that HF arise in response to the parasympathetic system stimulation, which is active during relaxing situations (Awais et al., 2017; Forcolin et al., 2018). HRV metrics is usually computed as the ratio between LF and HF (Pankaj et al., 2021), therefore it can be interpreted as a measure reflecting the balance between sympathetic and parasympathetic branches of the autonomous nervous system. In the present study, no significant variation of HRV-related metrics was observed, suggesting that the driving task did not induce a detectable modulation of the autonomous nervous system activity. An increase in heart rate (HR, Figure 10a) was observed during high fatigue compared to low fatigue periods independently from the driving environment, but again only if adopting the EEG-driven approach. Previous studies reported an increase of HR during cognitively demanding tasks, often interpreted as a marker of increased task engagement (Kennedy and Scholey, 2000; Darnell and Krieg, 2019). In this context, the increase in HR may reflect a compensatory physiological response to maintain performance as fatigue onset emerges. The absence of HRV modulation could be attributed to the relatively short duration of the task, designed to induce only mild fatigue onset rather than a deeply fatigued state. Thus, the HR increase without accompanying HRV changes may indicate heightened cognitive effort to counteract the initial effects of fatigue.

To summarize the findings highlighted, adopting a ToT-driven approach in defining fatigue intensity during a 45-min-long task performed in both simulated and real driving conditions, no physiological response to fatigue onset was observed. In contrast, adopting an EEG-driven approach, a physiological response to experiencing fatigue in the two environments, simulated and real, emerged. Considering this finding together with the results of subjective ratings of participants who reported an increase of experienced fatigue after the driving task, it appears that adopting the EEG-driven approach allowed to detect physiological traces of fatigue onset, which the ToT-driven approach failed to detect. It has to be noted that the results are similar between the two approaches in terms of trends, the difference mainly consisted of the effect size, consequently leading to statistically significant evidence when using the EEG-driven approach.

This finding answered the first research question (RQ1) posed in the present manuscript. It was demonstrated that, when dealing with early symptoms of fatigue, i.e., fatigue onset in a relatively short duration task, the ToT-driven approach is not sensitive enough towards the small physiological reactions to the investigated phenomenon. On the other hand, the EEG-estimated approach showed higher sensitivity, providing convincing evidence for its potential in fatigue detection compared to the conventional ToT-estimated approach. Nevertheless, further studies are needed to clarify the relationship between fatigue development, neurophysiological responses, and performance deterioration. In this regard, it would be advisable to adopt the MDrow index in less complex experimental settings, since the complexity of a real driving task may introduce additional noise into the investigation.

The secondary aim of this study (RQ2) was to explore eventual differences in experiencing fatigue in a simulated setting compared to a real driving task. To the best of authors’ knowledge, no prior study performed such a rigid controlled comparison, using identical participants and driving tasks across conditions. Subjective reports, and specifically the KSS, suggest that participants experienced fatigue in both simulated and real-world settings. Segmenting the data with an EEG-driven approach revealed that some of the physiological parameters considered showed different dynamics between simulated and real driving. Specifically, eyeblink duration and amplitude showed opposite dynamic to fatigue onset in the two different environments. Among heart-related parameters, no difference was observed between simulated and real driving fatigue onset. HR was found to increase during high fatigue both in simulated and real setting, while the other metrics were not affected either by fatigue levels or driving task. These results support the adoption of an objective physiological benchmark to define fatigue conditions in an unobtrusive manner.

If validated by future studies, adopting this approach offers key advantages. First, it will be possible to continuously monitor the driver, without any need for their input (as the case for subjective reports). One could argue that today, there are already some measures that can be used to continuously infer the mental state of a driver, for example, the driving performance. While driving and cognitive performance are often used as a proxy for mental states, behavioral signs typically emerge only after fatigue has already compromised task execution, potentially too late to prevent risk. Second, unlike the conventional ToT-driven approach, the EEG-based method adopted here could detect the onset of fatigue in a relatively short driving task, allowing for earlier and more effective intervention. Third, EEG-based fatigue detection can be used to label physiological, behavioral, and environmental data in order to train AI models in building a larger framework for understanding fatigue in operative contexts. Last but not least, in increasingly automated vehicles, the driver is often disengaged from physical control for extended periods.

Despite this, the driver must remain “in the loop” and ready to intervene in critical transitions. In such cases, behavioural cues become sparse or even unavailable, making them unreliable indicators of the driver’s readiness. Therefore, continuous and objective monitoring of the driver’s internal state through neurophysiological indicators becomes not only advantageous but essential.

Despite the relevance of these findings, several limitations should be acknowledged. Future implementations of this investigation should include a larger and more gender-balanced sample. Initially, 14 males and one female were recruited. However, the female participant was excluded from the final analysis to maintain a homogeneous sample. Although the sample used for the present study reflects the gender imbalance in the Italian and Spanish transport sector, it limits the generalizability of the findings to the broader population. Also, a key aspect of the present study is represented by the involvement of professional drivers. This choice was made thinking about a translational approach of the proposed method in real world. Indeed, professional drivers are a category which is strongly impacted by mental fatigue while driving, However, we acknowledge that professional drivers represent a subgroup of the general population. Therefore, before extending the present findings to the entire population, eventual bias due to the profession of the drivers should be accounted (professional vs. non-professional). Another limitation concerns the fixed order of task administration: participants always completed the simulated driving before the real driving. This decision was made to minimize safety risks associated with inducing fatigue in real-world driving, but it may have introduced order effects that should be considered when interpreting the results. Lastly, some variability was present between the experiments conducted at the two locations. With the current analytical design, it was not possible to fully account for potential biases arising from differences in location, vehicle, driving task, or experimenters. Future studies should explicitly model these factors in the analysis to better control for their possible influence on the results.

## Data Availability

The datasets presented in this study can be found in online repositories. The names of the repository/repositories and accession number(s) can be found below: https://zenodo.org/communities/fitdrive_h2020/records?q&equals;&l&equals;list&p&equals;1&s&equals;10&sort&equals;newest.
